# Diabetes status affects long-term changes in coronal caries - The SHIP Study

**DOI:** 10.1038/s41598-019-51086-z

**Published:** 2019-10-30

**Authors:** Julia Schmolinsky, Thomas Kocher, Wolfgang Rathmann, Henry Völzke, Christiane Pink, Birte Holtfreter

**Affiliations:** 1 Unit of Periodontology, Department of Restorative Dentistry, Periodontology, Endodontology, and Preventive and Pediatric Dentistry, University Medicine Greifswald, Greifswald, Germany; 2 German Diabetes Center, Institute for Biometrics and Epidemiology, Düsseldorf, Germany; 3 Institute for Community Medicine, University Medicine Greifswald, Greifswald, Germany; 4 German Centre of Diabetes Research, site Greifswald, Greifswald, Germany

**Keywords:** Dental caries, Risk factors

## Abstract

We estimated effects of diabetes mellitus and metabolic control on long-term change in coronal caries and restorative status using 11-year-follow-up data from the population-based Study of Health in Pomerania. Data of 3731 participants with baseline and 5- and 11-year follow-up information were included. Diabetes was defined via self-reported physician´s diagnosis or intake of glucose-lowering drugs or hemoglobin A1c (HbA1c) ≥6.5% or fasting blood glucose levels ≥11.1 mmol/l. The diabetes status was defined as no diabetes (HbA1c < 6.5% or non-fasting blood glucose <11.1 mmol/l), subjects with known or undetected diabetes mellitus and HbA1c ≤ 7% (well-controlled diabetes), and subjects with known or undetected diabetes mellitus and HbA1c > 7% (poorly-controlled diabetes). The caries status was clinically assessed using the half-mouth method and the Decayed Missing Filled Surfaces (DMFS) index and its component scores were determined. Covariate-adjusted linear mixed models were evaluated. Rates in change in DMFS were significantly higher in subjects with poorly-controlled diabetes compared to subjects without diabetes. Subjects with poorly- and well-controlled diabetes had significantly higher rates in change in Missing Surfaces (MS) compared to subjects without diabetes. For the DFS, rates in change were significantly lower for subjects with well-controlled diabetes and higher for subjects with poorly-controlled diabetes as compared to subjects without diabetes. Concordantly, all rates in change increased proportional to HbA1c levels. Effects were even more pronounced in subjects with diabetes duration of ≥5 years. Subjects with poorly-controlled diabetes are at higher risk for caries progression compared to subjects without diabetes, especially in case of longer disease duration.

## Introduction

Currently, diabetes mellitus (DM) has a global prevalence of 8.8% affecting 415 million adults and its prevalence is assumed to increase to 10.4% (642 million people) by 2040^1^. For Europe, Germany had the second-highest number of people with DM (6.5 million). DM is a metabolic disorder of multiple etiologies which leads to chronic hyperglycemia by defects in insulin secretion, insulin action, or both^2^. DM type 2 ranges from predominantly insulin resistance with relative insulin deficiency to a predominantly secretory defect with or without insulin resistance.

Dental caries is a process which results from an imbalance between re- and demineralization of the tooth substance caused by acidophilic oral microorganisms in the presence of high amounts of sugar and starch substrata^3^. In caries susceptible subjects a decreased flow rate, a lower pH-value and a lower mineral composition in saliva are associated with a lower remineralization. Indeed, these caries enhancing saliva properties are further impaired in patients with DM type 2 as compared to controls without diabetes^4^. Thus, an association between DM and coronal caries might be conceivable.

To date, there is no clear evidence that dental caries is related to DM. Previously published clinical studies were predominantly small, cross-sectional in nature and reported inconclusive results. While some reported higher levels in numbers of decayed, missing, or filled surfaces or teeth (DMFS or DMFT index) or its components in patients with diabetes compared to people without diabetes^4–11^, others found no significant differences^12–20^. Besides, only few studies dealt with the issue whether blood sugar control has an impact on caries^7–10,21^, with scattered positive findings in small clinical studies^7,8,10^. The population-based Korean National Health and Nutrition Examination Survey reported a significantly higher prevalence of untreated caries in subjects with uncontrolled diabetes compared to metabolically healthy subjects^21^. However, most of these clinical studies associating diabetes with caries were of low quality, high bias susceptibility, and with total sample sizes less than 300 subjects, suggesting that most studies were potentially underpowered. Consequently, well-designed large-scaled clinical and epidemiological studies are needed to improve evidence grade.

Thus, we investigated long-term effects of the diabetes status and hemoglobin A1c (HbA1c) levels on rates of change in coronal caries variables using 11-year follow-up data from the Study of Health in Pomerania (SHIP). We applied mixed models, which have greater power with continuous outcomes compared to linear regression models and explicitly incorporate inter-individual and intra-individual changes^22^. To estimate long-term effects, interaction terms between exposure and time are estimated; they correspond to exposure-dependent long-term changes in outcome variables. Importantly, this is the first investigation using prospective large-scaled population-based data.

## Materials and Methods

### Study of Health in Pomerania

SHIP is a population-based study in northeast Germany with baseline examinations conducted between 1997 and 2001^23^. A two-stage cluster sampling was adopted. First, of the three districts, three cities and twelve towns were selected, and of the small towns (<1500 inhabitants), 17 out of 97 were drawn at random. Second, from each of the selected communities the subjects were drawn at random, proportional to the population size and stratified by age and gender. In Greifswald, Stralsund, Anklam and 29 communities in the surrounding region a sample of 7006 individuals was drawn, aged 20 – 79 years, with 292 of each gender in each of the 12 five-year age strata. There were 741 neutral dropouts (126 had died, 615 had moved away) and five had severe medical problems. Of these 6265 eligible subjects, 4308 finally participated in the baseline study (SHIP-0), including 2193 women, corresponding to a response of 68.8%. The 5-year follow-up (SHIP-1) was conducted between 2002 and 2006 with 3,300 participants. Between 2008 and 2012, the 11-year follow-up with 2333 participants was conducted (SHIP-2). The study protocol was approved a priori by the Ethics Committee of the University of Greifswald, and written informed consent was obtained from each participant. The study was performed in accordance with relevant guidelines and regulations.

### Caries examination

Carious defects, fillings, secondary caries, and missing teeth were registered by surface (occlusal, mesial, distal, vestibular, oral) with the exception of wisdom teeth. Because full-mouth examinations are too time-consuming, subjects were examined according to the half-mouth method on the right or left side in alternate subjects. This is reasonable as the intra-oral distribution of caries can assumed to be symmetrical between the left and right side^24,25^. Coronal caries was identified visually using a periodontal probe (SHIP-0/2: PCP-11; SHIP-1: PCP-2; Hu-Friedy, Chicago, IL, USA) according to the WHO criteria^26^. To define coronal caries experience, the decayed missing filled surfaces (DFMS) index, the number of decayed or filled surfaces (DFS), and the number of missing surfaces (MS) were determined. It should be noted, that coronal caries was recorded according to the half-mouth method. Thus, scores stated in this manuscript need to be doubled if compared to other studies that applied full-mouth recordings. To prevent bias associated with tooth extraction when calculating the DFS, surfaces were reduced to those retained over all three examinations.

Dental examinations were performed by trained and licensed dentists. During the course of the study, calibration exercises were held every 6 to 12 months on persons not related to the study. For SHIP-0, intra- and inter-examiner agreement was 100%. For SHIP-1 and SHIP-2, intra-rater kappas were 0.95–0.99 and 0.83–1.00, respectively, while pairwise inter-rater kappas were 0.91–0.99 and 0.72–1.00, respectively.

### Periodontal examination in SHIP-0

Probing depth (PD) and clinical attachment levels (CAL) were recorded at four sites per tooth (distobuccal, mesiobuccal, midbuccal, midpalatinal/midlingual) using a periodontal probe (PCP-11, Hu-Friedy, Chicago, IL, USA). Measurements were taken according to the half-mouth method alternatingly on the left and right side, excluding third molars, and mathematically rounded to the nearest millimeter.

### Assessment of the diabetes status

HbA1c was measured by high performance liquid chromatography (SHIP-0: Diamat, Bio-Rad 7 Laboratories, Hercules, California, U.S.A.; SHIP-1: ClinRep HbA1C, Recipe Chemicals Instruments GmbH, Munich, Germany; SHIP-2: Diamat Analyzer; Bio-Rad, Munich, Germany). In SHIP-0 and SHIP-1, non-fasting serum glucose was measured with a Hitachi 717 analyzer (Roche, Mannheim, Germany) while in SHIP-2 a Dimension Vista 500 analytical system (Siemens AG, Erlangen, Germany) was used. Known or detected diabetes mellitus was defined as self-reported physician’s diagnosis or intake of glucose-lowering drugs (Anatomical Therapeutic Chemical Classification System (ATC) code A10) or glycated hemoglobin (HbA1c) concentrations of ≥6.5% (≥47.54 mmol/mol)^27^ or non-fasting serum glucose >11.1 mmol/l^28^. Duration of known diabetes mellitus (defined as self-reported physician’s diagnosis or intake of glucose-lowering drugs (ATC code A10)) was categorized as less than or at least five years according to information from the interview.

As exposure variables, i) a categorical variable referred to as ‘diabetes status’ combining information on known or detected diabetes mellitus with HbA1c levels: no known or detected diabetes mellitus (no diabetes), known or detected diabetes mellitus with HbA1c ≤ 7% (well-controlled diabetes), or known or detected diabetes mellitus with HbA1c > 7% (poorly-controlled diabetes) and ii) continuous HbA1c levels while adjusting for known diabetes mellitus were employed.

### Covariate measurements

Baseline behavioral and socioeconomic data were retrieved from a computer-assisted interview. School education was defined as <10/10/>10 years. Smoking status was defined as never/former/current smoking. Information on dental visit within the last 12 months, tooth brushing frequency (<versus ≥2 times/day), and the use of interdental care devices (flossing, dental sticks, tooth picks, interdental brushes) were collected. Waist circumference was measured to the nearest 0.1 cm using an inelastic tape.

### Study sample

**Figure 1:**
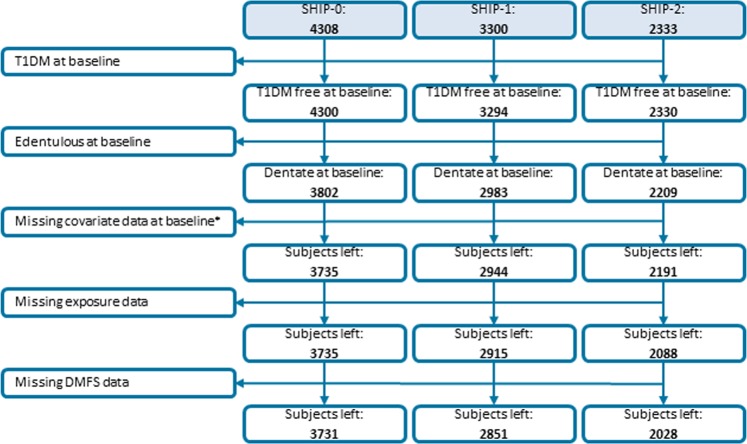
Flowchart showing exclusion criteria and number and type of missing data for the DMFS sample. SHIP, Study of Health in Pomerania; T1DM, Type 1 diabetes mellitus; DMFS, Decayed, missing, filled surfaces. *For at least one covariate.

Subjects with type 1 diabetes mellitus at baseline (N = 8), subjects being edentulous at baseline (N = 498) and subjects with missing covariate data at baseline (N = 67) were excluded (Fig. 1). Then, considering each of the three examinations separately, subjects with either missing data for the diabetes status or the DMFS were excluded. Finally, for each examination, 3731, 2851, and 2028 subjects were left for analyses, respectively.

### Statistical analyses

Means with standard deviations (SDs) and, if appropriate, medians with 25% and 75% quantiles were reported for continuous variables. Relative frequency distributions were computed for categorical variables. Chi-squared tests or Kruskal-Wallis-tests were conducted to detect distributional differences across groups defined by diabetes status.

Linear mixed effects models (subject, time) with random intercepts and slopes for time across subjects, and robust standard errors were conducted to estimate longitudinal effects of exposure variables on outcome variables^29–31^. Mixed models utilize all available data across visits and handle missing data flexibly^30^. Time-varying levels of outcome variables were modelled as dependent variables. For all models the fixed factors part included baseline levels for confounders, as well as baseline and follow-up visit levels of exposure variables, follow-up time, and the interaction term between the exposure variable and time^31^. By including the interaction term between the exposure variable and time, exposure-dependent differences in rates of change in kidney function variables over time are identified^31^. Examination times (referred to as ‘time’) were determined as exact years (with decimals) from start of SHIP-0. Based on prior clinical knowledge, confounders included age (modelled as restricted cubic splines with four knots), gender, school education, smoking, partnership, waist circumference (modelled linearly), dental visit within the last 12 months, interdental cleaning, and tooth brushing frequency.

Linear regression coefficients (B) or odds ratios (OR) with 95% confidence intervals (CIs) were reported. Interaction terms between the exposure variable and time can be interpreted as exposure-dependent differences in rates of change in caries variables over time^31^. Post-hoc linear combinations of coefficients for rates of change (surfaces per year) were calculated. For graphical illustration, model-based predictions (fixed part only) of outcome levels across fixed levels of exposure variables while adjusting for remaining variables in the model were calculated. Effect modifications by age and gender were tested and found to be non-significant. As sensitivity analyses, all models were repeated with stratification for duration of diabetes (<5 and ≥5 years).

Two-sided p values < 0.05 were considered statistically significant. All analyses were performed using Stata/SE 14.2^32^ and R 3.4.4^33^.

## Results

### Subject characteristics

At baseline (SHIP-0), 3402 subjects had no diabetes, while 185 and 144 had well or poorly controlled diabetes, respectively (Table 1). In 68.9% and 79.8% of subjects with well-controlled or poorly controlled diabetes, respectively, diabetes durations of ≥5 years were observed. Mean follow-up time since start of SHIP-0 was 12 years in all three groups. Subjects with diabetes were older, more often male, less often highly educated, less often current smokers, and more often presented with central adiposity compared to subjects without diabetes. At SHIP-1 and SHIP-2, percentages of edentulous subjects with diabetes (irrespective of their HbA1c level) were doubled compared to subjects without diabetes (p < 0.05 for SHIP-1). All caries variables differed significantly between the three groups for all examinations (p < 0.05). For subjects with diabetes (irrespective of their HbA1c level) highest levels of DMFS and MS, but lowest levels of DFS were observed. However, it should be noticed that i) numbers of subjects differed across examinations and ii) variable distributions within groups were highly confounded by, for example, age.

**Table 1:** Baseline characteristics according to baseline diabetes status (N = 3731); Study of Health in Pomerania.

	N total	Baseline diabetes status		
		Subjects without diabetes	Subjects with well-controlled diabetes	Subjects with poorly controlled diabetes
**Baseline covariates**				
N	3731	3402	185	144
Age, years	3731	46.3 ± 15.1	63.9 ± 10.7	60.4 ± 11.0*
Male gender	3731	47.6%	61.1%	62.5%*
Known diabetes mellitus (yes)	3731	—	66.5%	79.9%*
HbA1c, %	3731	5.2 ± 0.5	6.3 ± 0.7	8.5 ± 1.2*
HbA1c, mmol/mol	3731	33.3 ± 5.9	45.0 ± 7.3	69.2 ± 13.3*
Duration of diabetes (subjects with diabetes only)	233			
<5 years		—	31.1%	20.2%
≥5 years		—	68.9%	79.8%
School education	3731			
<10 years		31.1%	66.0%	69.4%
10 years		50.3%	24.3%	18.8%
>10 years		18.6%	9.7%	11.8%*
Smoking status	3731			
Never smoker		35.7%	36.7%	39.6%
Ex-smoker		31.3%	44.3%	46.5%
Current smoker		33.0%	18.9%	13.9%*
Partnership (yes)	3731	76.6%	77.8%	77.8%
Waist circumference, cm	3731	87.4 ± 13.6	99.1 ± 12.5	101.9 ± 14.0*
Dental visit within the last 12 months (yes)	3731	89.4%	88.1%	81.9%*
Tooth brushing frequency ≥2 times/day (yes)	3731	84.3%	71.4%	66.7%*
Interdental care (yes)	3731	36.0%	22.2%	26.4%*
Mean probing depth, mm	3686	2.49 ± 0.71	2.87 ± 0.99	3.08 ± 1.05*
Mean attachment level, mm	3521	2.47 ± 1.80	4.12 ± 1.95	4.34 ± 2.08*
**Edentulism (yes)**				
SHIP-1	2851	1.8%	5.3%	6.0%*
SHIP-2	2028	3.4%	6.5%	6.5%
**Time since start of SHIP-0**, **years**				
SHIP-0	3731	1.5 ± 0.8	1.5 ± 0.8	1.4 ± 0.9
SHIP-1	2851	6.7 ± 1.0	6.7 ± 0.9	6.5 ± 1.0
SHIP-2	2028	12.4 ± 1.2	12.5 ± 1.1	12.4 ± 1.3
**DMFS**				
SHIP-0	3731	32.0 ± 16.9	43.5 ± 14.9	41.0 ± 17.2*
SHIP-1	2851	34.2 ± 16.9	42.7 ± 17.1	44.4 ± 16.5*
SHIP-2	2028	35.8 ± 16.6	42.8 ± 16.9	41.0 ± 17.0*
**MS**				
SHIP-0	3731	17.2 ± 17.3	32.9 ± 18.5	32.7 ± 18.7
		10 (5; 26)	33 (15; 51)	34 (17; 50)*
SHIP-1	2851	18.1 ± 17.9	31.3 ± 20.7	35.0 ± 19.6
		10 (5; 28)	28 (15; 52)	35 (20; 52)*
SHIP-2	2028	18.0 ± 18.0	28.7 ± 20.3	27.3 ± 21.4
		10 (5; 25)	25 (10; 47)	24 (9; 47)*
DFS				
SHIP-0	3731	13.5 ± 9.1	9.6 ± 9.0	6.8 ± 6.8*
SHIP-1	2789	15.6 ± 9.3	11.2 ± 9.1	9.4 ± 7.9*
SHIP-2	1938	18.5 ± 9.6	15.6 ± 9.5	14.8 ± 9.2*
**Number of teeth**				
SHIP-0	3731	20.6 ± 7.3 23 (17; 26)	14.0 ± 8.1 15 (6; 22)	13.9 ± 8.1 13 (7; 21)*
SHIP-1	2851	20.2 ± 7.6 23 (17; 26)	14.5 ± 8.8 17 (6; 22)	13.0 ± 8.8 13 (5; 21)*
SHIP-2	2026	20.2 ± 7.6 23 (17; 26)	15.7 ± 8.5 18 (9; 23)	16.1 ± 9.3 18 (8; 24)*

Data are presented as mean ± standard deviation, partly with median (25% quantile; 75% quantile) or row percentages. DMFS, Decayed missing filled surfaces index; MS, number of missing surfaces; DFS, Number of decayed or filled surfaces. *p < 0.05 (Chi-squared test or Kruskal-Wallis test) for comparison across groups defined by diabetes status within each SHIP examination.

If stratified by age (Table 2), differences between groups were still evident and MS, DFS and the number of teeth differed significantly between the three groups for the two older age groups (p < 0.05). For subjects with poorly controlled diabetes highest levels of MS, but lowest levels of DFS were observed (due to the lower number of exposed surfaces). Note that the number of 20–39-year-olds with diabetes was too low to detect any differences.

**Table 2:** Baseline levels of caries indices and components stratified by age and baseline diabetes status (N = 3731); Study of Health in Pomerania.

	Baseline diabetes status		
	Subjects without diabetes (N = 3402)	Subjects with well-controlled diabetes (N = 185)	Subjects with poorly controlled diabetes (N = 144)
**N**			
20–39 years	1327	4	4
40–59 years	1351	56	62
60–81 years	724	125	78
**DMFS**			
20–39 years	22.6 ± 13.8	25.8 ± 9.0	19.5 ± 18.0
40–59 years	34.2 ± 15.1	37.1 ± 15.4	34.2 ± 17.4
60–81 years	45.2 ± 14.9	46.9 ± 13.6	47.4 ± 14.0
**MS**			
20–39 years	6.8 ± 9.4 5 (0; 10)	9.8 ± 9.5 5 (5; 14.5)	8.8 ± 8.5 7.5 (2.5; 15)
40–59 years	18.3 ± 15.4 15 (5; 25)	22.2 ± 17.1 20 (10; 31)	25.2 ± 18.5 20 (10; 41)*
60–81 years	34.0 ± 17.8 34.5 (20; 50)	38.5 ± 16.6 42 (25; 52)	39.9 ± 15.7 44 (29; 52)*
**DFS**			
20–39 years	14.9 ± 8.7	15.5 ± 4.4	10.8 ± 10.4
40–59 years	14.1 ± 9.4	12.9 ± 10.2	7.4 ± 6.9*
60–81 years	9.7 ± 8.3	7.9 ± 8.0	6.1 ± 6.5*
**Number of teeth**			
20–39 years	24.9 ± 3.8 26 (24; 28)	24 ± 3.6 25 (21.5; 26.5)	24.3 ± 2.5 24.5 (22.5; 26)
40–59 years	20.2 ± 6.4 22 (17; 25)	18.9 ± 6.9 21 (16.5; 23)	17.0 ± 8.0 19 (11; 24)*
60–81 years	13.3 ± 7.8 13 (6; 20)	11.5 ± 7.5 10 (6; 17)	10.8 ± 7.0 10 (5; 15)*

Data are presented as mean ± standard deviation, partly with median (25% quantile; 75% quantile). DMFS, Decayed missing filled surfaces index; MS, number of missing surfaces; DFS, Number of decayed or filled surfaces. *p < 0.05 (Kruskal-Wallis test) for comparison across groups defined by diabetes status within each age stratum.

### Effects of diabetes status on long-term change in caries variables

**Figure 2:**
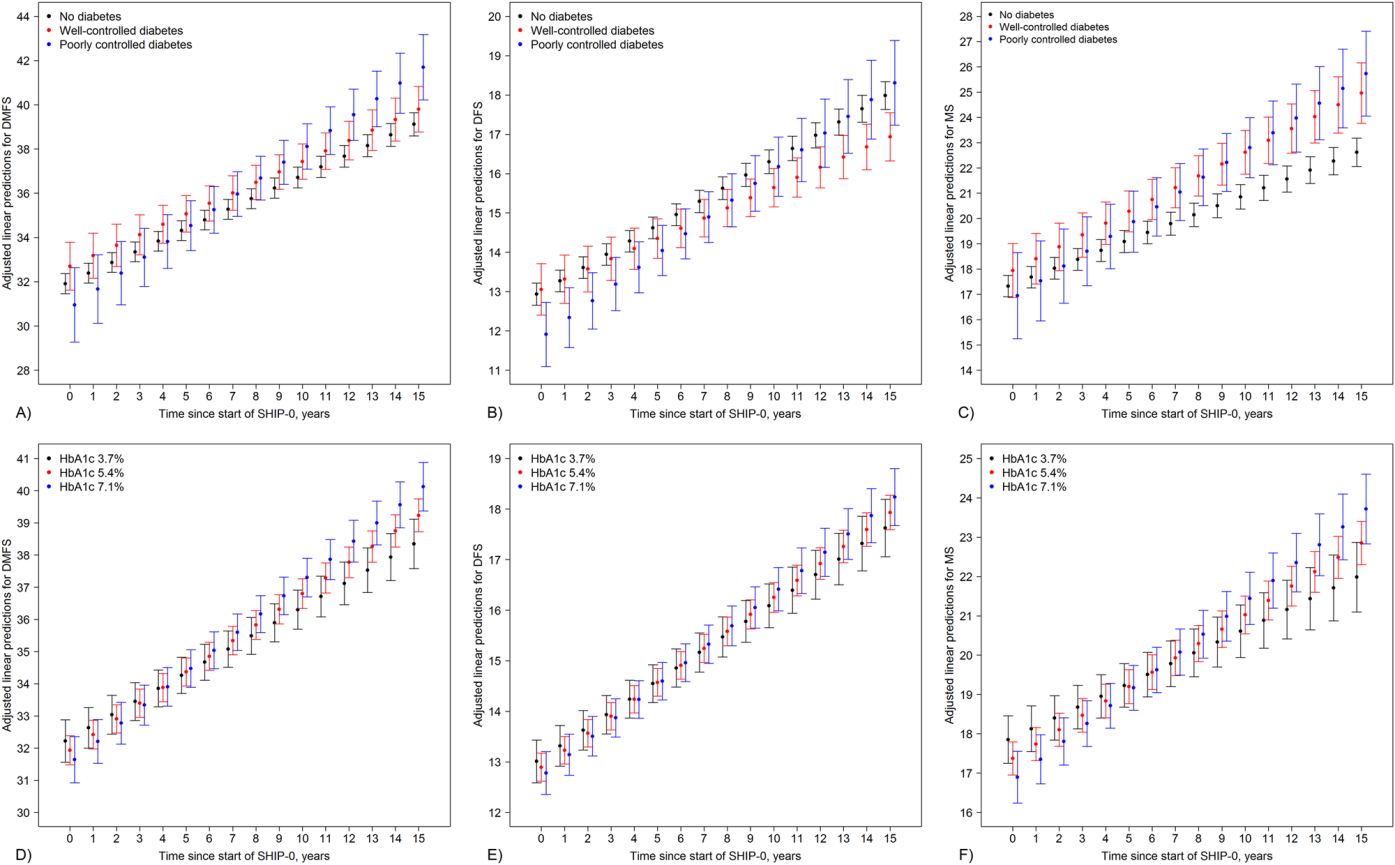
In the upper part, model predicted DMFS (A; including all surfaces), DFS (B; including only retained surfaces), and MS (C; including all surfaces) over time since start of study for subjects without diabetes (black), subjects with well-controlled diabetes (red), and subjects with poorly controlled diabetes (blue) are shown. In the lower part, model predicted DMFS (D; including all surfaces), DFS (E; including only retained surfaces), and MS (F; including all surfaces) over time since start of study for subjects with an HbA1c of 3.7% (mean – standard deviation; black), 5.4% (mean; red), and 7.1% (mean + standard deviation; blue) are shown. Model predicted means (only fixed effects) and confidence intervals are given.

Long-term changes in caries variables differed significantly according to diabetes status (Table 3 and Fig. 2A–C). At the initial status, no significant differences compared to subjects without diabetes were found (Table 3 and Fig. 2A). But with 0.716 surfaces per year for the DMFS index (95% CI 0.552 to 0.880), only subjects with poorly controlled diabetes had statistically relevantly higher DMFS progression rates compared to subjects without diabetes (0.473 surfaces per year, 95% CI 0.378 to 0.569), but also compared to subjects with well-controlled diabetes (0.473 surfaces per year, 95% CI 0.378 to 0.569; p = 0.01 from post-hoc tests). Subjects with well-controlled diabetes neither showed significant differences in the initial status nor in rates of change compared to subjects without diabetes.

**Table 3:** Results from linear mixed models evaluating long-term effects of diabetes status on the DMFS index, the DFS, and the MS component.

DMFS	Total sample		Including only diabetes patients with diabetes duration ≥5 years		Including only diabetes patients with diabetes duration ≥5 years	
	B (95% CI)	P value	B (95% CI)	P value	B (95% CI)	P value
N_SHIP-0_/N_SHIP-1_/N_SHIP-2_	3731/2851/2028		3671/2755/1937		3558/2692/1892	
***Linear mixed model (fixed part only)***						
Diabetes status (ref. no diabetes)						
Well-controlled diabetes	0.792 (−0.249; 1.833)	0.14	0.350 (−0.886; 1.586)	0.58	0.834 (−0.607; 2.276)	0.26
Poorly controlled diabetes	−0.957 (−2.632; 0.718)	0.26	−0.942 (−2.929; 1.046)	0.35	−0.112 (−3.030; 2.806)	0.94
Time since start of SHIP-0, years	0.480 (0.454; 0.507)	<0.001	0.479 (0.452; 0.505)	<0.001	0.481 (0.454; 0.507)	<0.001
Interaction between diabetes status and time		0.02*		0.03*		
Well-controlled diabetes X Time	−0.007 (−0.106; 0.092)	0.89	0.047 (−0.083; 0.178)	0.48	−0.008 (−0.160; 0.144)	0.92
Poorly controlled diabetes X Time	0.236 (0.069; 0.402)	0.006	0.264 (0.065; 0.463)	0.009	0.089 (−0.187; 0.366)	0.53
***Post-hoc linear combinations of coefficients for rates of change (surfaces per year)***						
No diabetes	0.480 (0.454; 0.507)		0.479 (0.452; 0.505)		0.481 (0.454; 0.507)	
Well-controlled diabetes	0.473 (0.378; 0.569)		0.526 (0.399; 0.654)		0.472 (0.322; 0.623)	
Poorly controlled diabetes	0.716 (0.552; 0.880)		0.743 (0.546; 0.939)		0.570 (0.295; 0.845)	
**DFS**						
N_SHIP-0_/N_SHIP-1_/N_SHIP-2_	3731/2789/1938		3671/2697/1858		3558/2638/1812	
***Linear mixed model (fixed part only)***						
**Diabetes status (ref**. **no diabetes)**						
Well-controlled diabetes	0.121 (−0.510; 0.752)	0.71	−0.037 (−0.795; 0.721)	0.92	0.834 (−0.607; 2.276)	0.26
Poorly controlled diabetes	−1.024 (−1.837; −0.211)	0.01	−1.774 (−2.772; −0.776)	<0.001	−0.112 (−3.030; 2.806)	0.94
Time since start of SHIP-0, years	0.337 (0.318; 0.356)	<0.001	0.337 (0.318; 0.356)	<0.001	0.481 (0.454; 0.507)	<0.001
Interaction between diabetes status and time		0.005*		0.005*		
Well-controlled diabetes X Time	−0.078 (−0.136; −0.020)	0.009	−0.082 (−0.156; −0.009)	0.03	−0.008 (−0.160; 0.144)	0.92
Poorly controlled diabetes X Time	0.090 (−0.005; 0.184)	0.06	0.137 (0.022; 0.252)	0.02	0.089 (−0.187; 0.366)	0.53
***Post-hoc linear combinations of coefficients for rates of change (surfaces per year)***						
No diabetes	0.337 (0.318; 0.356)		0.337 (0.318; 0.356)		0.481 (0.454; 0.507)	
Well-controlled diabetes	0.259 (0.203; 0.315)		0.255 (0.184; 0.326)		0.298 (0.205; 0.392)	
Poorly controlled diabetes	0.426 (0.334; 0.519)		0.474 (0.361; 0.587)		0.467 (0.289; 0.645)	
**MS**						
N_SHIP-0_/N_SHIP-1_/N_SHIP-2_	3731/2851/2028		3671/2755/1937		3558/2692/1892	
***Linear mixed model (fixed part only)***						
**Diabetes status (ref**. **no diabetes)**						
Well-controlled diabetes	0.618 (−0.406; 1.642)	0.24	0.438 (−0.789; 1.666)	0.48	0.456 (−0.934; 1.846)	0.52
Poorly controlled diabetes	−0.380 (−2.095; 1.335)	0.66	0.247 (−1.745; 2.238)	0.81	0.012 (−3.640; 3.664)	0.99
Time since start of SHIP-0, years	0.353 (0.328; 0.378)	<0.001	0.351 (0.326; 0.376)	<0.001	0.352 (0.327; 0.378)	<0.001
Interaction between diabetes status and time		0.005*		0.03*		
Well-controlled diabetes X Time	0.115 (0.005; 0.225)	0.04	0.137 (−0.006; 0.279)	0.06	0.129 (−0.039; 0.297)	0.13
Poorly controlled diabetes X Time	0.233 (0.061; 0.404)	0.008	0.186 (−0.006; 0.377)	0.06	0.097 (−0.236; 0.430)	0.57
***Post-hoc linear combinations of coefficients for rates of change (surfaces per year)***						
No diabetes	0.353 (0.328; 0.378)		0.351 (0.326; 0.376)		0.352 (0.327; 0.378)	
Well-controlled diabetes	0.468 (0.360; 0.576)		0.488 (0.348; 0.628)		0.481 (0.313; 0.649)	
Poorly controlled diabetes	0.586 (0.417; 0.755)		0.537 (0.347; 0.726)		0.450 (0.118; 0.781)	

Linear mixed models (subjects, time) with random intercepts and random slopes for time, and robust standard errors were applied. Models were adjusted for baseline levels of age (cubic splines with 4 knots), gender, school education, smoking, dental visits, interdental cleaning, tooth brushing frequency, partnership, and waist circumference as fixed effects. For exposure, time, and outcome variables, baseline and follow-up levels were included. For easy interpretation of rates of change according to diabetes status, post-hoc linear combinations of coefficients were calculated and presented below respective models. DMFS, Decayed missing filled surfaces index; DFS, Decayed filled surfaces; MS, Missing surfaces; B, linear regression coefficient; CI, confidence interval. *p values from Chi^2^-tests testing the joint hypothesis that B = 0 for all respective indicator variables.

Considering the DFS (Table 3 and Fig. 2B), subjects with poorly controlled diabetes had significantly lower DFS values compared to subjects without diabetes at the initial status (B = −1.024, 95% CI −1.837 to −0.211). Compared to subjects without diabetes (0.337 surfaces per year, 95% CI 0.318 to 0.356) rates of change were significantly (p = 0.009) lower for subjects with well-controlled diabetes (0.259 surfaces per year, 95% CI 0.203 to 0.315), but only borderline significantly (p = 0.06) higher for subjects with poorly controlled diabetes (0.426 surfaces per year, 95% CI 0.334 to 0.519). Differences in rates of change between subjects with well- and poorly controlled diabetes were statistically significant (p = 0.003; from post-hoc tests).

For the MS component (Table 3 and Fig. 2C), baseline MS levels did not differ significantly for subjects with diabetes compared to subjects without diabetes. Regarding rates of change, subjects with poorly controlled diabetes (0.586 surfaces per year, 95% CI 0.417 to 0.755; p = 0.008) and subjects with well-controlled diabetes (0.468 surfaces per year, 95% CI 0.360 to 0.576; p = 0.04) had significantly higher rates of change compared to subjects without diabetes (0.353 surfaces per year, 95% CI 0.328 to 0.378). Differences in rates of change between subjects with well- and poorly controlled diabetes were statistically non-significant (p = 0.24; from post-hoc tests).

When subjects with diabetes were restricted to those with diabetes durations of ≥5 years, differences in rates of change were generally more pronounced. With 0.743 surfaces per year for the DMFS index (95% CI 0.546 to 0.939), subjects with poorly controlled diabetes had relevantly higher DMFS progression rates compared to subjects without diabetes. For the DFS, rates of change were significantly lower for subjects with well-controlled (0.255 surfaces per year, 95% CI 0.184 to 0.326) and significantly higher for subjects with poorly controlled diabetes (0.474 surfaces per year, 95% CI 0.361 to 0.587) compared to subjects without diabetes (0.337 surfaces per year, 95% CI 0.318 to 0.356). For the MS component, rates of change were higher (though with borderline significance; p = 0.06) for subjects with well-controlled (0.488 surfaces per year, 95% CI 0.348 to 0.628) and poorly controlled diabetes (0.537 surfaces per year, 95% CI 0.437 to 0.726) compared to subjects without diabetes (0.351 surfaces per year, 95% CI 0.326 to 0.376).

When subjects with diabetes were restricted to those with a duration of diabetes of <5 years (Table 3), differences in rates of change were smaller and did not differ significantly between groups for all three caries variables.

### HbA1c and long-term change in caries variables

Evaluation of linear effects of continuously modeled HbA1c levels on caries variables (Table 4 and Fig. 2D–F) revealed that rates of change increased proportional to increasing HbA1c levels for the DMFS index (B = 0.046, 95% CI 0.014 to 0.078) and the MS component (B = 0.053, 95% CI 0.017 to 0.088), i.e. the higher the HbA1c level, the higher the rate in change in both the DMFS index and the MS component was. In detail, for an HbA1c of 3.7, 5.4, and 7.1%, the DMFS progressed by 0.408, 0.487, and 0.566 surfaces per year, respectively (Fig. 2D). Accordingly, for an HbA1c of 3.7, 5.4, and 7.1%, the MS component progressed by 0.275, 0.365, 0.455 surfaces per year, respectively (Fig. 2F). Considering the DFS, no statistically significant differences in rates of change according to HbA1c levels were seen (p = 0.15, Table 4 and Fig. 2E).

**Table 4:** Results from linear mixed models evaluating long-term effects of HbA1c levels (continuously modelled) on the DMFS index, the DFS, and the MS component.

DMFS	Total sample		Including only diabetes patients with diabetes duration ≥5 years		Including only diabetes patients with diabetes duration <5 years	
	B (95% CI)	P value	B (95% CI)	P value	B (95% CI)	P value
N_SHIP-0_/N_SHIP-1_/N_SHIP-2_	3716/2848/2022		3656/2752/1931		3543/2692/1886	
HbA1c, %	−0.170 (−0.476; 0.135)	0.28	−0.188 (−0.520; 0.144)	0.27	−0.054 (−0.421; 0.312)	0.77
Time since start of SHIP-0, years	0.237 (0.064; 0.409)	0.007	0.172 (−0.016; 0.359)	0.07	0.334 (0.128; 0.541)	0.001
**Interaction between HbA1c and time**						
HbA1c X Time	0.046 (0.014; 0.078)	0.005	0.059 (0.024; 0.094)	0.001	0.027 (−0.011; 0.066)	0.17
**DFS**	**B (95% CI)**	**P value**				
N_SHIP-0_/N_SHIP-1_/N_SHIP-2_	3716/2786/1932		3656/2694/1852		3543/2692/1886	
HbA1c, %	−0.068 (−0.256; 0.120)	0.48	−0.133 (−0.342; 0.076)	0.21	−0.018 (−0.255; 0.219)	0.88
Time since start of SHIP-0, years	0.247 (0.125; 0.368)	<0.001	0.194 (0.063; 0.326)	0.004	0.255 (0.092; 0.418)	0.002
**Interaction between HbA1c and time**						
HbA1c X Time	0.016 (−0.006; 0.039)	0.15	0.027 (0.002; 0.051)	0.03	0.015 (−0.015; 0.046)	0.33
**MS**	**B (95% CI)**	**P value**				
N_SHIP-0_/N_SHIP-1_/N_SHIP-2_	3716/2848/2022		3656/2752/1931		3543/2692/1886	
HbA1c, %	−0.281 (−0.557; −0.006)	0.045	−0.258 (−0.551; 0.036)	0.09	−0.272 (−0.611; 0.068)	0.12
Time since start of SHIP-0, years	0.080 (−0.110; 0.270)	0.41	0.059 (−0.144; 0.263)	0.57	0.142 (−0.087; 0.372)	0.22
**Interaction between HbA1c and time**						
HbA1c X Time	0.053 (0.017; 0.088)	0.003	0.056 (0.018; 0.094)	0.004	0.040 (−0.003; 0.083)	0.07

Linear mixed models (subjects, time) with random intercepts and random slopes for time, and robust standard errors were applied. Models were adjusted for baseline levels of age (cubic splines with 4 knots), gender, school education, smoking, dental visits, interdental cleaning, tooth brushing frequency, partnership, waist circumference, and known diabetes mellitus as fixed effects. For exposure, time, and outcome variables, baseline and follow-up levels were included. DMFS, Decayed missing filled surfaces index; DFS, Decayed filled surfaces; MS, Missing surfaces; B, linear regression coefficient; CI, confidence interval.

Restricting subjects with diabetes to those with duration of ≥5 years, differences in rates of change were more pronounced. Rates of change increased proportional to increasing HbA1c levels for the DMFS index (B = 0.059, 95% CI 0.024 to 0.094), the DFS (B = 0.027, 95% CI 0.002 to 0.051), and the MS component (B = 0.056, 95% CI 0.018 to 0.094).

## Discussion

To our knowledge, this is the first prospective large-scaled population-based study evaluating long-term effects of diabetes status and HbA1c levels on caries progression. Rates of change in the DMFS index and the MS component were significantly more pronounced in subjects with poorly controlled diabetes compared to subjects without diabetes; also rates of change in the DMFS index and the DFS were significantly more pronounced in subjects with poorly controlled diabetes compared to subjects with well-controlled diabetes. In addition, comparing subjects with well-controlled diabetes with subjects without diabetes, rates of change were less pronounced for the DFS. Also, progression rates were significantly higher in subjects with higher HbA1c levels for the DMFS index and the MS component utilizing the total sample, and also for the DFS in subjects with diabetes durations of ≥5 years. Taken together, our results confirm the hypothesis that diabetes status and metabolic control might affect long-term caries progression.

Only one population-based study differentiated between well and poorly controlled diabetes^21^, reporting consistent results. In the Korean National Health and Nutrition Examination Survey increasing levels of fasting blood glucose and HbA1c were associated with a 26% higher prevalence of untreated caries in people with uncontrolled diabetes compared to metabolically healthy people^21^. In small clinical studies metabolic control was related to at least one of the considered coronal caries indices or their single or combined components^7–10^. For example, an Indian study found highest DS or DMFT levels in patients with uncontrolled diabetes, while lowest DMFT levels were found in metabolically controlled diabetes patients^8^.

Different pathways may explain the association between poorly controlled DM and coronal caries. In patients with poorly controlled diabetes, a diet with high rates of sugar-containing food and drinks might lead to unfavorable local and systemic effects. Systemically, a sugar or starch rich diet leads to increased blood glucose levels, which in turn leads to increased levels of glucose in saliva and in gingival cervicular fluid^34–36^, which both bath the supragingival microbiome and trigger caries progression. Indeed, high blood glucose concentrations in patients with diabetes correlate with high salivary glucose concentrations^34–36^. Locally, sugar is metabolized by cariogenic bacteria, resulting in higher acid concentrations and, thus, more demineralization^6,8^. Usually a higher intake of oral sugar stimulates saliva flow, which eliminates some of the sugar from the oral cavity. However, diabetes patients have a reduced saliva flow rate in comparison to subjects without diabetes^6,8,37^. Thus, oral sugar clearance is disturbed in diabetes patients with uncontrolled diabetes. Taken together, more acidic saliva, decreased saliva flow rates, and lower mineral compositions in saliva are associated with a lower remineralization, thereby increasing the risk of dental caries. If metabolic dysbalances with increased HbA1c levels continue over several years, cariogenic processes accumulate, as seen in this study. Differences in rates of change for caries variables were generally more pronounced, when subjects with diabetes were restricted to those with diabetes durations of ≥5 years.

To put our results into a clinical perspective, the DMFS score needs some explanation: A molar has five and an incisor four surfaces (thus a score of 4 or 5 equals one tooth). Further, scores reported in this study have to be doubled because the caries status was assessed using a half-mouth examination. However, because of the bilateral symmetry of coronal caries indices^25,38^, it can be assumed that the half-mouth recording sufficiently reflects the full-mouth recording. Compared to subjects without diabetes, subjects with poorly controlled diabetes had about 0.5 (2 × 0.236) additional surfaces suffering from decay, filling or extraction each year, equating to about 1.5 teeth over a period of 15 years. On average, subjects with poorly controlled diabetes had 14 teeth at baseline, whereas subjects without diabetes had 21 teeth. Thus, an additionally missing tooth may endanger the support for the removable denture; at least it necessitates extension of an existing one. In this regard, the general physician and diabetologists should make diabetes patients with poor metabolic control aware of their potential tooth loss due caries and they should advise them to seek care and prophylaxis from the dentist. The treating dentist should more often perform caries preventive measures such as application of fluoride varnish to counteract the acidophilic attack. And last but not least, poorly controlled diabetes patients should be advised to improve personal oral hygiene with adjunctive fluoride gel or mouth rinses in addition to the general advice to improve their metabolic control through reduction of diabetes risk factors^39^.

In adults, most teeth are extracted either due to caries or due to periodontitis^40^. Thus the M component represents the endpoint of both oral diseases and it is unknown to what extent teeth were extracted due to caries or periodontitis. Thus, results for indices including the M component (i.e. DMFS and MS) should be interpreted with care. In a previous SHIP publication, we showed that over a 5-year period periodontitis progression was four times and incident tooth loss was two times higher in poorly controlled versus subjects with well-controlled diabetes or healthy subjects^41^. In the studied sample, baseline levels of mean PD and mean CAL differed significantly across groups with lowest levels found in subjects without diabetes and highest levels found in subjects with poorly controlled diabetes (Table 1). Thus, differences in rates of change in the DMFS index and the MS component related to the diabetes status and the level of metabolic control might partially be explained by chronic periodontitis driving tooth loss. However, it needs to be emphasized that rates of change in the DFS, which reflects the number of surfaces with active caries lesions and fillings, were significantly more pronounced in subjects with poorly controlled diabetes compared to subjects with well-controlled diabetes, indicating a potential relevance of the metabolic control level. Further, progression rates for the DFS were significantly higher in subjects with higher HbA1c levels, though only in subjects with diabetes durations of ≥5 years. Taken together, the DFS might also be associated to the level of metabolic control.

Another aspect observed in this study deserves consideration. Interestingly, subjects with well-controlled diabetes had lower rates in change in DFS compared to subjects without diabetes and subjects with poorly controlled diabetes (Table 3). Possibly, subjects with well-controlled diabetes might have a better general and possibly also oral health awareness, might practice better oral hygiene and care, might have regular medical control and consultations, and might pursue a balanced and sugar-reduced diet. However, baseline data only partially support these assumptions: compared to subjects with poorly controlled diabetes, subjects with well-controlled diabetes more often brushed their teeth at least twice a day (71.4 vs. 66.7%) and more often had visited their dentist within the last year (88.1 vs. 81.9%). However, subjects without diabetes performed even better for both aspects (84.3% and 89.4%, respectively). Finally, it remains speculative, why subjects with well-controlled diabetes had lower incidence rates for active caries lesions and fillings in this study.

Some strengths of our study merit special consideration. First, this is the first prospective large-scaled population-based study evaluating long-term effects of diabetes status and HbA1c levels on coronal caries experience covering an average follow-up time of 11 years. Thus, in contrast to previously published clinical studies, results can be generalized to the adult Caucasian population. Second, dental examiners were well-trained and calibrated before and during baseline and follow-up examinations. Third, we applied mixed models, which can handle subject-specific time intervals and unbalanced data, thus making use of all available data in parameter estimation^29–31^, thereby limiting also selection bias through built-in imputation methods in case of examination-wise incomplete data. Further, mixed models explicitly account for inter-individual and intra-individual change and estimate long-term effects via evaluation of interaction terms between exposure variables and time. Coefficients for these interaction terms correspond to exposure-dependent differences in rates of change in caries variables. Further, since time was continuously modeled, power for detecting time dependent effects was increased^42^. Lastly, multilevel models are flexible in specifying the variance-covariance structure and inclusion of time-varying variables.

Nevertheless, it should be noted that, when interpreting our results, linking of time-varying exposures and outcomes in the presence of reciprocal causation might have converted our longitudinal problem into a cross-sectional one^31^. However, because coronal caries does not affect metabolic status, we can exclude the possibility of reciprocal causation and interpret results as long-term effects of metabolic status on caries progression.

Further limitations of our study also need to be considered. Firstly, the half-mouth caries recording might have led to an underestimation of caries severity estimates by about 50% with dilution of effect estimates towards the null^43^. Secondly, the clinical examination underestimates proximal caries in comparison to visual inspection and radiography^44^. Thus, effects of diabetes and metabolic control on caries were probably underestimated in our study.

Thirdly, changes in diabetes status and HbA1c levels were assessed at only three time points, rather than multiple time points, giving only a rough picture of changes in the metabolic status. Lastly, because fluoride and sugar intake were not assessed in SHIP, we could not account for potential confounding effects of both factors. Systemic intake of fluoride is probably more or less evenly distributed in the studied population with intake of fluoridated common salt being common, but with no systemic fluoride administration (e.g. water fluoridation or fluoride tablets); contribution to confounding is probably low. However, some minor confounding might have been introduced by application of adjunctive fluoride gels or mouth rinses. For sugar intake, no assumptions can be made and, thus, potential confounding of unknown extent cannot be excluded.

In summary, this is the first prospective large-scaled population-based study evaluating long-term effects of diabetes status and HbA1c levels on variables quantifying different aspects of coronal caries experience. Both diabetes status and HbA1c levels were consistently associated with the DMFS, the MS component and the DFS, with more pronounced effects if subjects with diabetes were restricted to those with diabetes durations of 5 years, thus confirming previously published smaller clinical studies. Even though effects of diabetes status and HbA1c levels on the DFMS and the MS component were probably partially explained by periodontitis-related tooth loss, we need to emphasize that the number of surfaces with active caries lesions and fillings was significantly related to the level of metabolic control. This supports the assumption that metabolic control might not only be related to periodontitis, but also to coronal caries. In any case, consequences for the diabetes patient are the same: the diabetes patient must become aware of the oral problem and more prophylactic home and professional measures should be initiated.

## Data Availability

Due to restrictions related to participant consent, all relevant data are available upon request. More information is available at the following http://www.medizin.uni-greifswald.de/cm/fv/ship.
